# A Case of Puerperal Eclampsia

**Published:** 1880-01

**Authors:** F. W. Bartlett


### Clinical Reports.

---

### A CASE OF PUERPERAL ECLAMPSIA.

REPORTED BY F. W. BARTLETT, M. D.

October 18th, 1879, I was summoned hastily to attend Mrs. C., primipara, aged 26 years, for whom I had made an obstetric engagement; unable to leave another patient, I referred the case to Dr. J. W. Keene; upon visiting the patient at midnight, Dr. K. found no evidences of labor; patient had headache, nausea, steady unremitting pain in abdomen, which she thought had been induced by fatigue, having been very busily employed in household duties during the day. Squibb's tinct. opium was ordered in ten-drop doses; after about two hours' delay Dr. K. returned home; at 2:30 A. M. the 19th, half an hour after leaving the patient, he was again called to find her slowly recovering from a convulsion; Dr. A. Dagenais, who had been summoned, was also present. In answer to another summons, being at liberty, I proceeded to the house, entering just as a second convulsion occurred. The violence of the convulsion was subdued by etherization, and we consulted as to the proper course to pursue.

It may be stated here that I had six weeks before tested for albumen with a negative result. Two weeks later, however, it was notably present, and saline cathartics and usual diuretics were ordered, which failed to lessen the proportion of albumen; the patient was plethoric and œdematous; sixteen ounces of blood were taken from the arm on October 12th, seven days prior to confinement.

After interchange of views, I examined and found the os uteri unchanged; no evidence of dilatation; Dr. Keene administered thirty drops of fluid extract ergot hypodermically, and fifteen minutes later fifteen grains chloral hydrate in same manner. This plan was followed until about six doses of each article had been injected at intervals of fifteen minutes each; meanwhile, I practiced manual dilatation, introducing one, two and ultimately three fingers within the os, until it was dilated to about one and a half inches diameter. I then passed the right blade of Prof. White's forceps, and after a vexatious but necessary delay succeeded in introducing the left, and locked them. The cervical opening did not then exceed two inches in diameter, and the os was still thick and rigid.

Traction was carefully made, and at the expiration of an hour, or about 6:30 A. M., three hours after dilatation was attempted, the delivery was accomplished—child still-born. At 8:30 a strong convulsion; injected fifteen grains of chloral; chloroform by inhalation; convulsions occurred every two hours until 5:30 P. M., when they ceased entirely. Catheterization at 10:30 A. M.,; bladder mpty. At 4 P. M., urine 12 ounces, solid albumen by heat and nitric acid; at 10 P. M., 16 ounces, one-fourth albumen by estimate; urine was taken by catheter for five days; only a trace of albumen after second day; consciousness fully restored on fourth day; patient is now, eight weeks elapsing, in perfect health. Three of the hypodermic punctures produced painful and tedious abscesses; careful examination of cervix, urethra and periænum prove them uninjured.

Special facts in history of patient: her mother died at term of puerperal convulsions—mother's sister also of same; grandmother, maternal side, of same; and mother's niece, cousin of Mrs. C., died also of same—a somewhat remarkable history!

Points of treatment: the prompt dilatation of the os by digital manipulations; hypodermic use of chloral and ergot; disuse of opium in any form; continuous anæsthesia by ether and chloroform.

Anticipatory treatment: cathartics, diuretics, venesection.

Reflections:—Was venesection useful and would repetition of same after convulsions be advisable? Does opium relieve or increase cerebral congestion in albuminuria? Does ergot protect, by its hemastatic quality, the brain from hemorrhage or effusion? What is the special influence of chloral upon convulsive phenomena?

I gratefully acknowledge my obligation to Drs. J. W. Keene and A. Dagenais for valuable counsel and co-operation in the management of the case.

